# Viral Diversity and Its Relationship With Environmental Factors at the Surface and Deep Sea of Prydz Bay, Antarctica

**DOI:** 10.3389/fmicb.2018.02981

**Published:** 2018-12-03

**Authors:** Zheng Gong, Yantao Liang, Min Wang, Yong Jiang, Qingwei Yang, Jun Xia, Xinhao Zhou, Siyuan You, Chen Gao, Jian Wang, Jianfeng He, Hongbing Shao, Andrew McMinn

**Affiliations:** ^1^College of Marine Life Sciences, Ocean University of China, Qingdao, China; ^2^Key Laboratory of Biofuels, Shandong Provincial Key Laboratory of Energy Genetics, Qingdao Institute of Bioenergy and Bioprocess Technology, Chinese Academy of Sciences, Qingdao, China; ^3^Key Lab of Polar Oceanography and Global Ocean Change, Ocean University of China, Qingdao, China; ^4^Institute of Evolution and Marine Biodiversity, Ocean University of China, Qingdao, China; ^5^SOA Key Laboratory for Polar Science, Polar Research Institute of China, Shanghai, China; ^6^Institute for Marine and Antarctic Studies, University of Tasmania, Hobart, TAS, Australia

**Keywords:** marine viruses, Prydz Bay, metagenomics, diversity, community structure

## Abstract

A viral metagenomic analysis of five surface and two bottom water (878 meters below surface, mbs, and 3,357 mbs) samples from Prydz Bay, was conducted during February–March 2015. The results demonstrated that most of the DNA viruses were dsDNA viruses (79.73–94.06%, except at PBI1, 37.51%). Of these, Caudovirales (*Siphoviridae, Myoviridae*, and *Podoviridae*) phages were most abundant in surface seawater (67.67–71.99%), while nucleocytoplasmic large DNA viruses (NCLDVs) (*Phycodnaviridae, Mimiviridae*, and *Pandoraviridae* accounted for >30% of dsDNA viruses) were most abundant in the bottom water (3,357 mbs). Of the ssDNA viruses, *Microviridae* was the dominant family in PBI2, PBI3, PBOs, and PBI4b (57.09–87.55%), while *Inoviridae* (58.16%) was the dominant family in PBI1. *Cellulophaga* phages (phi38:1 and phi10:1) and *Flavobacterium* phage 11b, infecting the possible host strains affiliated with the family *Flavobacteriaceae* of the phylum *Bacteroidetes*, were abundant in surface water dsDNA viromes. The long contig (PBI2_1_C) from the viral metagenomes were most similar to the genome architectures of *Cellulophaga* phage phi10:1 and *Flavobacterium* phage 11b from the Arctic Ocean. Comparative analysis showed that the surface viral community of Prydz Bay could be clearly separated from other marine and freshwater environments. The deep sea viral community was similar to the deep sea viral metagenome at A Long-term Oligotrophic Habitat Assessment Station (ALOHA, at 22°45′N, 158°00′W). The multivariable analysis indicated that nutrients probably played an important role in shaping the local viral community structure. This study revealed the preliminary characteristics of the viral community in Prydz Bay, from both the surface and the deep sea. It provided evidence of the relationships between the virome and the environment in Prydz Bay and provided the first data from the deep sea viral community of the Southern Ocean.

## Introduction

Viruses are the most abundant and genetically diverse acellular biological entities in the ocean and play significant roles in microbial mortality, fluctuations in microbial community structure, and the horizontal gene transfer of genetic diversity between host cells (Wommack and Colwell, 2000; Breitbart et al., 2002; Weinbauer, 2004; Suttle, 2005, 2007). Although new culture-independent methods, such as metagenomics and single-cell genomics, have advanced the understanding of bacterial and archaeal diversity (Sharon and Banfield, 2013; Lasken and McLean, 2014), the diversity and variability of natural viral communities is still not well documented, mostly because of the lack of universal gene markers for viral communities and the relatively recent development and application of new culture-independent methods based on high-throughput sequencing to investigate viral diversity in different environments (Brum et al., 2015; Aylward et al., 2017).

Since the first marine viral metagenomic research in 2002 (Breitbart et al., 2002), metagenomics has become as a powerful tool to better understand viral diversity and variability and it can also be used to directly resolve complicated, uncultured environmental viral genomes (Angly et al., 2006; Culley et al., 2006; Hurwitz and Sullivan, 2013). Recently, viral metagenomic surveys in the Tara Ocean expeditions identified a total of 15,222 epipelagic and mesopelagic dsDNA viral populations, representing 867 viral clusters (approximately at genus-level groups) (Brum et al., 2015; Roux et al., 2016a). Virome information has now been recovered from many different marine environments through metagenomics (Breitbart et al., 2002; Angly et al., 2006; Steward and Preston, 2011; Williamson et al., 2012; Hurwitz and Sullivan, 2013; Brum et al., 2015).

The Southern Ocean, which connects the Pacific Ocean, the Indian Ocean and the Atlantic Ocean, plays an important role in global marine ecosystems and climate change and acts as a global biological and chemical transport channel (Evans and Brussaard, 2012; Wu et al., 2016). However, there are still very few reports on viral diversity in the Southern Ocean. Currently, there have only been two Southern Ocean viral DNA and RNA metagenomic studies and these are from the coastal waters of the Palmer Long-Term Ecological Research (PAL-LTER) Station B and Western Antarctic Peninsula (WAP) (Brum et al., 2016; Miranda et al., 2016). Temperate viruses were found to dominate the DNA viruses of the PAL-LTER site and there was a switch from lysogeny to lytic replication as the bacterial production increased. The WAP DNA viral assemblages were genetically distinct from those of lower-latitude assemblages, primarily driven by the temperate viral dominance (Brum et al., 2016). It was found that RNA viruses contributed up to 65% of the total virioplankton (8–65%) and this is consistent with the hypothesis that RNA viruses influence diatom bloom dynamics in WAP coastal waters (Miranda et al., 2016). These analyses revealed the diversity and seasonal variations of the dsDNA and RNA viral communities in WAP coastal waters, respectively. However, our knowledge of the marine pelagic viral community, viral diversity and their correlations with environmental factors within the Southern Ocean is still quite limited, especially in the deep sea (>1,000 meters below surface, mbs).

Prydz Bay is the third largest embayment along the Antarctic margin and is located in the Indian Ocean section of the Southern Ocean, between 66°E and 79°E adjacent to the Amery Ice Shelf. On the continental shelf the water depth is mostly between 400 and 600 m and increases to the north to depths >3,000 m (Roden et al., 2013; Liang et al., 2016). There have been numerous marine microbial ecology studies in Prydz Bay (Waters et al., 2000; Pearce et al., 2007, 2010; Thomson et al., 2010), however, there are still few studies on the marine viral ecology (Paterson and Laybourn-Parry, 2012; Liang et al., 2016). There have still been no metagenomics studies of the viral community structure and diversity in Prydz Bay. We present here a viral metagenomic dataset, including five surface and two bottom seawater samples from Prydz Bay, including both double-stranded DNA (dsDNA), and single-stranded DNA (ssDNA). This study will provide the first data on the viral diversity, community structure, novel viruses and the relationship between viral communities and their environment in Prydz Bay, Antarctic.

## Materials and methods

### Sampling and environmental factors

**Figure 1:**
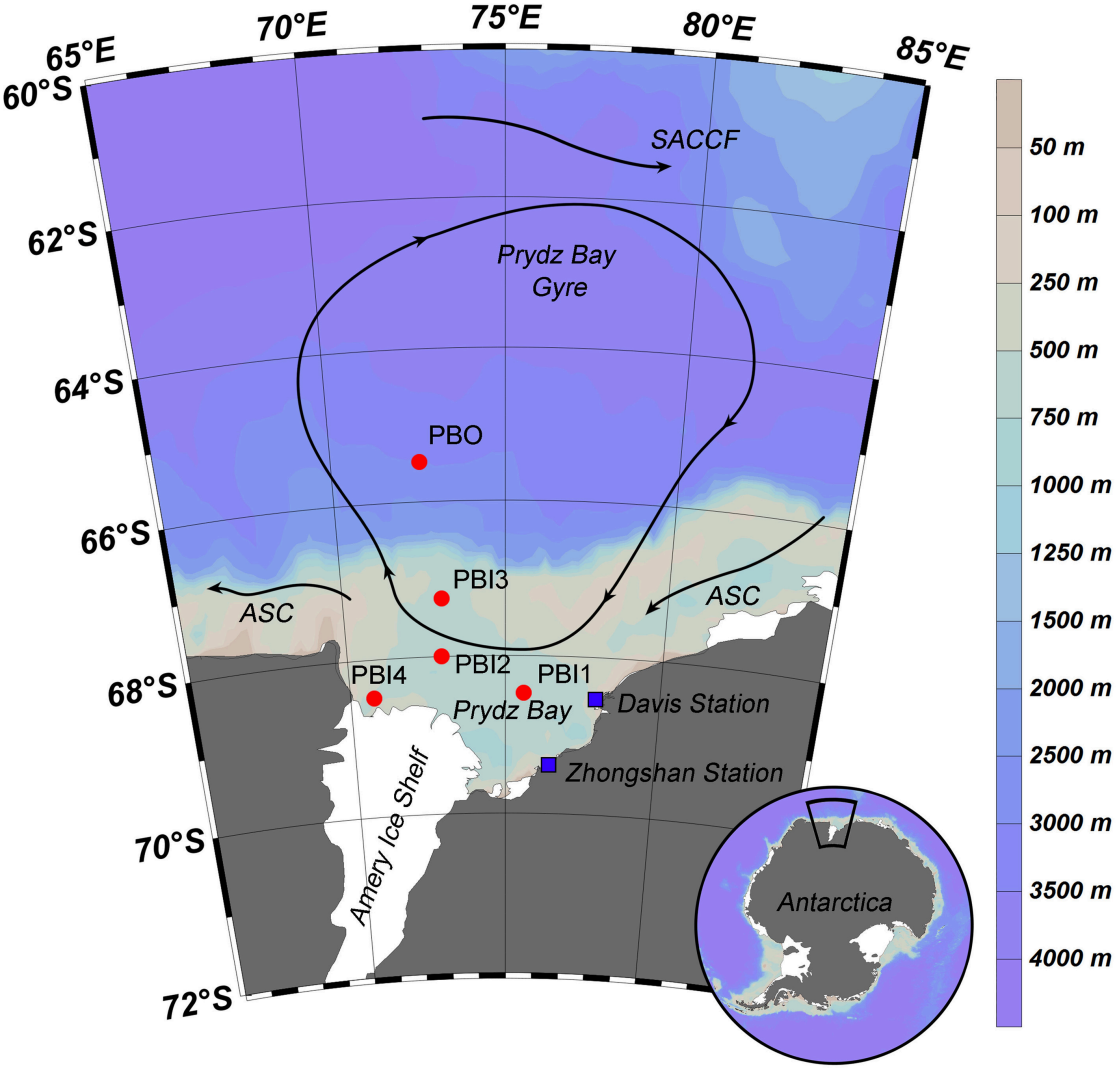
Sampling stations and summer water currents (Revised from Roden et al., 2013) in the Prydz Bay, Antarctic. The direction of the current (arrow and solid line), location of each sampling site (red point), and Antarctic scientific investigation station (blue block) were shown in the map. Different colors represent as deep gray (land), light gray—light blue—deep blue (the depth from shallow sea to deep sea). SACCF, Southern Antarctic Circumpolar Current Front; ASC, the Antarctic slope current.

Seven viral metagenomic seawater samples were collected from five stations in Prydz Bay and the adjacent Southern Ocean, during the 31st Chinese Antarctic Scientific Expedition from February 4th to March 2nd, 2015. The five stations were located in three different environments. Only surface samples were collected from stations PBI1, PBI2, and PBI3, which were located in inner Prydz Bay. Both surface and bottom samples were collected from station PBI4 (PBI4s and PBI4b), which was located near the western edge of Amery Ice Shelf, and from station PBO (PBOs and PBOb), which was located in the open Southern Ocean (Figure 1).

Seawater samples (300 L) were collected using Niskin bottles mounted on a rosette frame which also held the SBE-9 plus CTD sensors (SBE 911; Sea-Bird Electronics) for temperature, salinity, dissolved oxygen (DO), chlorophyll *a*, and depth. One hundred milliliter sub-samples were taken from each site to determine nutrient concentrations; these were filtered through GF/F filters (Whatman). The filtrates were poisoned by adding saturated mercuric chloride (ca. 1.5 × 10^−3^ v/v) and then stored at 4°C until analysis within 2 weeks. Nutrient concentrations (SiO_4_-Si, PO_4_-P, NH_4_-N, NO_2_-N, and NO_3_-N) were measured with an onboard nutrient auto-analyzer (SKALAR SAN plus, Netherlands). The detailed site information is presented in Table 1.

**Table 1:** Main environmental factors of seven viral metagenomic samples in the Prydz Bay.

**Sample name**	**Sample Depth (mbs)**	**Temperature (°C)**	**Salinity (PSU)**	**DO (μmol/L)**	**Chl*a* (mg/L)**	**PO_4_-P (μmol/L)**	**NO_2_-N (μmol/L)**	**SiO_3_-Si (μmol/L)**	**NO_3_-N (μmol/L)**	**NH_4_-N (μmol/L)**
PBI1	5	−1.26	32.21	384.43	19.72	0.24	0.54	1.86	3.13	2.24
PBI2	5	0.94	32.89	368.99	7.45	0.16	0.10	1.36	3.50	1.49
PBI3	5	−0.34	32.63	381.31	19.16	0.21	0.56	6.27	9.82	0.60
PBI4s	5	−0.81	33.37	307.79	3.37	0.63	0.50	21.76	8.02	5.36
PBOs	5	0.20	33.40	344.39	0.08	1.01	0.26	52.76	23.74	0.37
PBI4b	878	−1.88	34.54	209.30	0.04	1.97	0.18	59.11	29.86	0.63
PBOb	3,357	−0.12	34.66	223.98	0.04	2.34	0.26	110.54	34.25	0.41

*mbs, meters below surface; Chla, chlorophyll a; DO, dissolved oxygen; PO_4_-P, phosphate; SiO_3_-Si, silicate; NO_2_-N, nitrite; NO_3_-N, nitrate; NH_4_-N, ammonium*.

### Preparation of viral concentrates and DNA extraction

The seawater samples (300 L) were immediately filtered using a 300 mm diameter cellulose membrane with a 3 μm pore size and then filtered through a 0.22 μm membrane, to remove the large organisms, such as zooplankton, phytoplankton, and bacteria. The free viruses in the filtrate were concentrated to a volume of 500 ml by the large-scale Tangential Flow Filtration (membrane package with a total surface area of 0.5 m^2^: Pellicon® 2 Cassette, Biomax® 50 kDa; polyethersulfone) and then were concentrated further to 10 ml in plastic micro-tube by small-scale Tangential Flow Filtration (membrane package with a total surface area of 50 cm^2^: Pellicon® XL Cassette, Biomax® 50 kDa; polyethersulfone) (Sun et al., 2014). After each sample concentration, the filtration membrane cassettes were cleaned by flushing with sufficient deionized water, followed by circulation cleaning with 0.1 N NaOH for at least 30 min. The viral concentrates were flash-frozen in liquid nitrogen and then stored at −80°C until processed (Brussaard, 2004; Winter et al., 2014). Though the recovery efficiency of each viral concentrates was not checked, the recovery efficiency averaged from 27 at the deep ocean samples to 41% at the estuary samples using the Tangential Flow Filtration with polyethersulfone membranes (Cai et al., 2015).

The viral concentrates were unfrozen and re-filtrated through 0.22 μm filters to remove any remaining cellular microorganisms, then precipitated using polyethylene glycol (PEG-8000) (10% w/v) and NaCl (0.6% w/v) followed by incubation at 4°C in the dark for 24 h. The mixed samples were centrifuged at 10,500 × g for 40 min at 4°C and suspended in 300 μl SM buffer. One hundred microliter (1 M) KCl solution was added followed by incubation on ice for 30 min (Colombet et al., 2007). Though the recovery efficiency of viruses for the PEG precipitation of each sample was not measured in this study, the reported recovery efficiency averaged 55% (range 28–81%) (Colombet et al., 2007). Centrifugation at 12,000 × g for 10 min at 4°C was used to obtain concentrated and purified free virus-like particles (VLPs). The VLPs were treated with proteinase K and 10% SDS at 56°C for 1 h. The viral DNA was extracted using the phenol/chloroform/isoamylol method and stored at −80°C until sequencing (Thurber et al., 2009).

### Virome libraries construction and sequencing

The viral DNA was amplified with QIAGEN® REPLI-g Mini Kit (phi-29 DNA polymerase), which used the whole-genome multiple displacement amplification (MDA) method, for 16 h in a thermal cycler, using multiple 50 μl reactions containing 100–200 ng of the isolated DNA as a template (Angly et al., 2006; Yilmaz et al., 2010). Library construction and sequencing were implemented by Novogene Bioinformatics Technology Co. Ltd (Beijing, China). Sequencing libraries were generated using NEBNext® Ultra™ DNA Library Prep Kit for Illumina, following the manufacturer's instructions. Briefly, an ultrasonic processor was used for carrying qualified DNA fragmentation (Insert Size: 250 bp), DNA fragments were then end-polished, A-tailed, ligated with sequencing adapters and PCR amplified. Finally, PCR products were purified (AMPure XP system) and the library's insert size was verified by Agilent 2100 Bioanalyzer and quantified using real-time PCR. High-throughput sequencing was performed by the Illumina HiSeq 4000 platform (Paired-End Sequencing, 2 × 150 bp).

### Metagenomic and genomic analyses

High-quality reads were selected from raw reads giving 47–94 million (clean data rate >0.90) 150 bp paired-end reads. The paired-end reads were filtered by adopting the following conditions: (1) contained more than 10% N; (2) were of a low quality (40% reads length, Q ≤ 5); (3) with the adapter. Quality-filtered reads were assembled using Velvet (version 1.2.10) (Zerbino and Birney, 2008; Zerbino et al., 2009). After assembling, the contigs with lengths < 300 bp were filtered out. A summary of the seven viromes is shown in Table 2. To determine the relative abundance of viral contigs, the quality-filtered reads from each metagenome were mapped back to the assembled contigs with Bowtie2 (version 2.1.0) (Langmead and Salzberg, 2012) and SAMtools (version: 1.1) (Li et al., 2009). The average abundance was calculated as DNA-reads per kilobase of the transcript (gene) per million reads mapped (DNA-RPKM; equal to the number of reads mapped to the contig and normalized by the contig length and per million mappable reads) (Calusinska et al., 2016).

**Table 2:** Data summary of seven viral metagenomic samples in the Prydz Bay.

**Sample name**	**Total length (kbp)**	**GC content (%)**	**Longest (bp)**	**N50**	**Average length (bp)**	**Number of contigs (>300 bp)**	**Affiliated contigs**	**Affiliated dsDNA viruses contigs**	**Affiliated ssDNA viruses contigs**	**Reads mapped**
PBI1	7,201,486	54.22	130,439	443	506	14,184	2,037	1,393	544	25,558,846 (34.31%)
PBI2	15,913,696	47.95	64,194	472	521	30,482	4,547	4,024	260	11,947,719 (25.54%)
PBI3	26,757,818	55.46	95,664	620	591	45,162	5,827	5,458	130	23,780,237 (29.88%)
PBI4s	895,331	50.75	20,377	420	460	1,938	256	235	3	2,188,596 (04.63%)
PBOs	10,755,196	53.93	40,732	444	479	22,366	2,315	2,053	131	17,433,201 (18.60%)
PBI4b	11,628,812	56.79	71,333	518	540	21,491	2,245	1,919	217	18,379,075 (31.99%)
PBOb	1,172,928	46.46	10,477	433	465	2,512	270	215	38	2,889,990 (04.84%)

*Affiliated contigs: contigs were assigned to be of viral origin based on LCA affiliation via MetaVir2. Reads mapped: number of reads mapped back to the assembled contigs (% of reads used for the assembly)*.

The assembled contigs were uploaded to the MetaVir2 server (Roux et al., 2014) (http://metavir-meb.univ-bpclermont.fr, project ID 8183, 8184, 8185, 8186, 8187, 8188, and 8189).

Taxonomic annotation: open reading frames (ORFs) were first predicted for each contig through MetaGeneAnnotator (Noguchi et al., 2008) and then compared to the RefSeq complete viral genomes protein sequence database from NCBI (release of 2017-01-11) using BLASTp (threshold of 50 on the BLAST bit score). Contigs with multiple ORFs were taxonomically assigned according to the lowest common ancestor (LCA) affiliation.

Genome reconstruction: circular contigs and linear contigs larger than 30 kb obtained from the assembly were examined further. Putative ORFs within contigs selected for further analysis were searched against the NCBI non-redundant protein database using BLASTp (E < 10^−3^). Comparisons with 40 other publicly available viromes (the details of the selected viromes are shown in Table S3) were based on k-mer frequency bias (tetranucleotides) (Willner et al., 2009). The non-normalized taxonomic composition data based on contigs best BLAST hit (threshold of 50 on the BLAST bit score) of PBOb (Project ID 8183) and ALOHA station deep abyss (Project ID 3816) were downloaded from MetaVir2 server and further for these two samples' comparison analysis.

Functional analysis of dsDNA viral communities was performed with Meta Genome Rapid Annotation using Subsystem Technology (MG-RAST) server (Meyer et al., 2008) (http://metagenomics.anl.gov/, MG-RAST ID 4747904.3, 4747905.3, 4747906.3, 4747909.3, 4747910.3, 4747911.3, and 4749478.3). The affiliated dsDNA virus contigs processed by MG-RAST were compared to the SEED Subsystems database using a maximum *E*-value of 10^−5^, a minimum identity of 60%, and a minimum alignment length of 15. A flowchart depicting the use of contigs was shown in Figure S1.

### Statistical analysis

dsDNA viral communities cluster analysis and statistical analysis were performed using PRIMER v5 (PRIMER E, Ltd, UK) (Relative abundance of virus in each sample represent the biont number). Canonical correspondence analysis (CCA) and redundancy analysis (RDA) were performed in R v. 3.5.1 (R Development Core Team) using CCA and RDA functions from the “vegan” package v2.5-2 (Oksanen et al., 2018) to investigate the relationships between viral species and environmental variables. A matrix of the total viral species (1,225 species) underwent factor analysis. A total of 10 environmental variables were used to assess the variation of viral species, including depth, salinity, temperature, DO, Chl*a*, NO_3_-N, NO_2_-N, NH_4_-N, PO_4_-P, and, SiO_3_-Si. All variables were logarithmically (base 10) transformed before CCA to reduce the influence of extreme values on ordination scores and to normalize data distribution.

### Accession number

All the viral reads data in this study were submitted to NCBI Sequence Read Achieve (SRA). The SRA accession number: SRP085759 (study).

## Results

### Environmental factors

The main environmental factors against which the seven viral metagenomic samples were correlated are shown in Table 1. Water temperature ranged from −1.88°C at 878 mbs to 0.94°C at the surface (PBI2). Chlorophyll *a* concentration in the surface samples (3.37–19.72 mg L^−1^) were naturally higher than in the bottom waters (0.04 mg L^−1^) except in PBOs (0.08 mg L^−1^). The salinity in the bottom samples (34.54–34.66) were a little higher than that at the surface (32.21–33.40) (P < 0.002). The DO in the bottom samples (209.30–223.98 μmol L^−1^) was lower than those at the surface (307.79–384.43 μmol L^−1^) (P < 0.002). The concentrations of PO_4_-P, SiO_3_-Si, and NO_3_-N in the adjacent Southern Ocean (PBOs and PBOb) and PBI4b were higher than that in other samples in the inner bay (P < 0.05).

The PBI4s and PBOb samples had some site-specific characteristics. PBI4s, which was apparently influenced by glacial meltwater, had high NH_4_-N but low DO and Chl*a* concentrations compared to other surface samples. PBOb had the highest PO_4_-P, SiO_3_-Si, and NO_3_-N concentrations.

According to the cluster analysis of environmental factors, two major groups were identified, group I (PBI1, PBI2, PBI3) and group II (PBOs, PBI4b, PBOb, and PB14s). PBI4s was separated from the other three samples within group II (Figure S2).

### Metavirome production and contig assembly

The assembled contigs data were analyzed using MetaVir2 and the statistical data of each sample can be seen in Table 2. A comparison of the ratio of affiliated contigs to unaffiliated contigs is shown in Figure S3. The number of contigs (>300 bp) varied from 1,938 at PBI4s to 45,162 at PBI3, with the average length being between 460 and 591 bp. Only 4.63–34.31% of the reads could be mapped back to the assembled contigs. PBI4s and PBOb had lower assembled contigs (1,938 and 2,512, respectively), affiliated contigs (256 and 270, respectively) and the ratios of mapped reads (4.63 and 4.84%, respectively) than other samples (ranging from 14,184 to 45,162, 2,037 to 5,827 and 18.60 to 34.31%, respectively) in Prydz Bay.

### Taxonomic composition of Prydz Bay dsDNA and ssDNA viral communities

**Figure 2:**
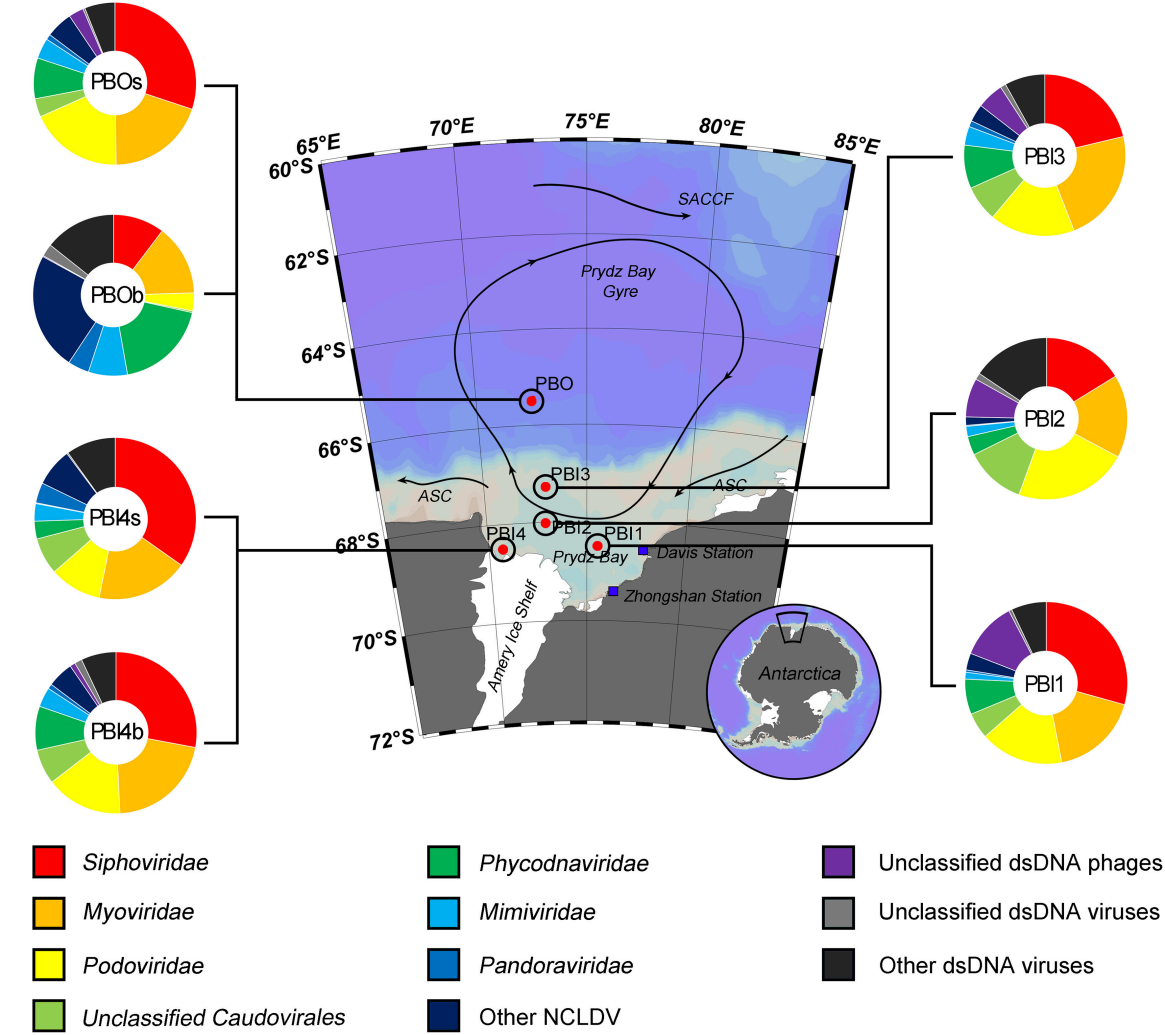
Taxonomic composition of annotated dsDNA viromes in the Prydz Bay, Antarctic. The pie charts show a global representation of dsDNA viral groups for all seven samples from the Prydz Bay. Different colors refer to different viral groups showed in the legend on the bottom.

Most of the contigs of the viral metagenomic data (85.08–89.65%) were unaffiliated; the majority of the affiliated contigs were affiliated to dsDNA viral contigs (68.38–93.67%, Figure S3). The most abundant viral groups [relative abundance after normalization (DNA-RPKM)] were dsDNA viruses (79.73–94.06%), except in PBI1 where they accounted for 37.51% (Figure S4). The order Caudovirales contained the most abundant dsDNA viruses in the surface samples and the bottom sample (PBI4b), which was collected from the edge of the Amery Ice Shelf (ranging from 67.67% at PBI2 to 71.99% at PBOs). Within the Caudovirales, *Siphoviridae* (29.34–34.78%) was more abundant than *Myoviridae* (17.60–21.30%) and *Podoviridae* (10.37–18.58%) (Figure 2). Interestingly, *Cellulophaga* phages and *Flavobacterium* phage 11b (NC_006356.2) were abundant in the surface dsDNA viromes. *Cellulophaga* phage phi38:1 (NC_021796.1) was most abundant and second most abundant in PBI2 (3.95%) and PBI3 (3.37%), respectively. *Cellulophaga* phage phi10:1 (NC_021802.1) was dominant in PBOs (6.44%). *Flavobacterium* phage 11b was abundant in PBOs (3.61%) and PBI2 (1.36%) (Table S1). Although both PBI4b and PBOb were collected from the bottom, there was a significant difference in the viral community structure between these two samples. The viral community in PBI4b was similar to the surface samples, but PBOb was clearly different from all other samples (Figure 2). In PBOb, while the proportion of Caudovirales (28.47%) was lower than in the other samples, the *Phycodnaviridae* (18.74%), *Mimiviridae* (7.84%), and *Pandoraviridae* (4.28%) families, which belong to the nucleocytoplasmic large DNA viruses (NCLDVs) and known to contain giant viruses infecting eukaryotes (Claverie et al., 2009), were much more abundant than in the other samples (Figure 2). Within the *Phycodnaviridae, Chrysochromulina ericina* virus (CeV, classified within genus *Prymnesiovirus*, 6.78%), *Aureococcus anophagefferens* virus (AaV, classified within genus *Phaeovirus*, 4.99%), *Ostreococcus lucimarinus* virus 2 (OlV2, classified within genus *Prasinovirus*, 2.23%), *Emiliania huxleyi* virus 86 (EhV86, classified within genus *Coccolithovirus*, 1.75%), and *Phaeocystis globosa* virus (PgV, classified within genus *Prymnesiovirus*, 0.84%) were dominant in PBOb. Within the *Mimiviridae, Acanthamoeba polyphaga moumouvirus* (ApMoV, 3.27%), *Acanthamoeba polyphaga mimivirus* (ApMiV, 2.66%), and *Cafeteria roenbergensis* virus BV-PW1 (CrV, 1.71%) were dominant in PBOb.

Regarding the main host classifications, *Cellulophaga* phages (around 4.93%), *Pseudomonas* phages (around 3.20%), and *Vibrio* phages (around 3.24%) were responsible for the high percentage in the surface samples. In the bottom samples, however, *Acanthamoeba polyphaga* viruses (5.93% for PBOb and 2.46% for PB4b) and Pandoravirus (4.28% for PBOb), which are giant viruses belonging to NCLDVs, comprised the largest proportion of the dsDNA viral community (Tables S1, S2).

**Figure 3:**
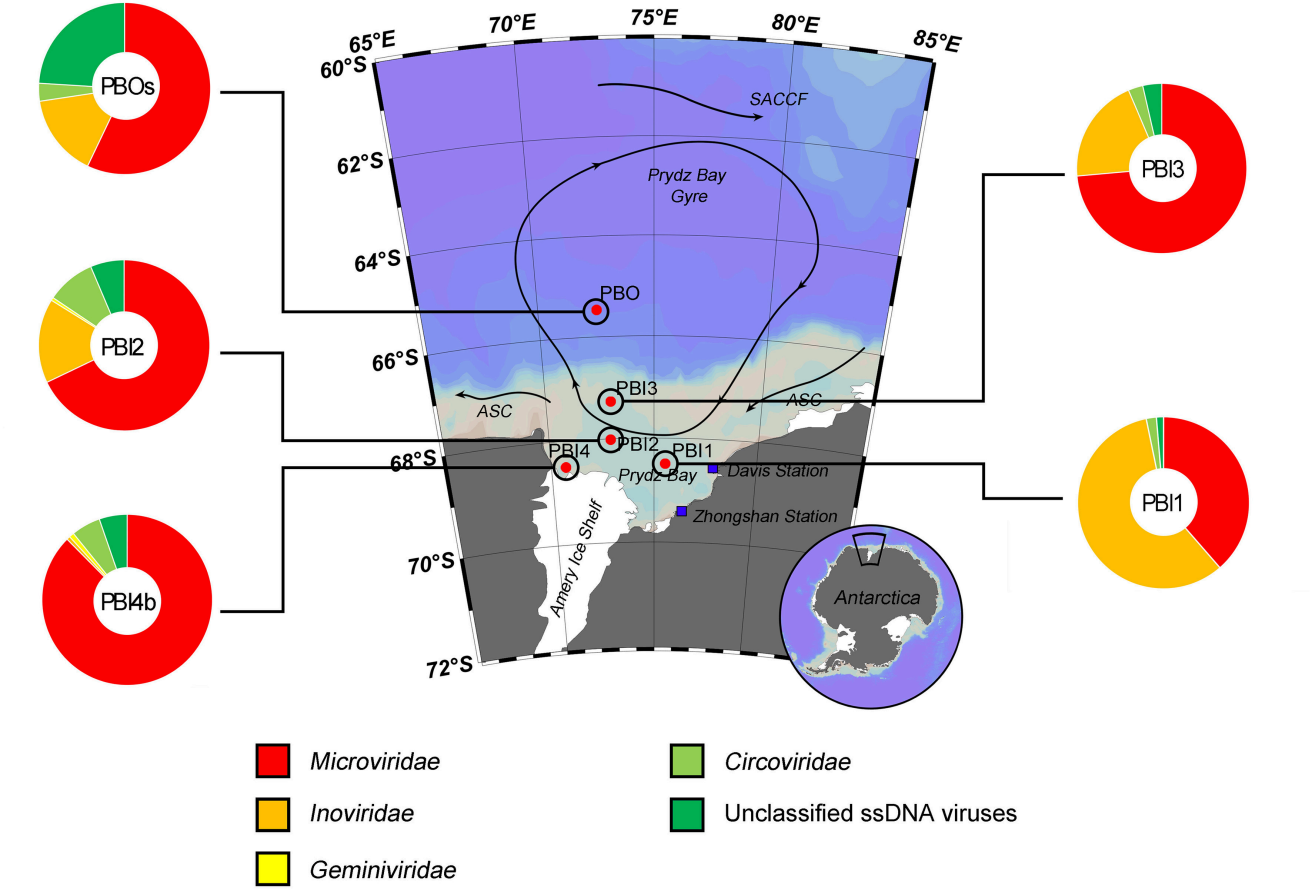
Taxonomic composition of annotated ssDNA viromes in the Prydz Bay, Antarctic. The pie charts show a global representation of ssDNA viral groups for five samples (except PBI4s and PBOb) from the Prydz Bay. Different colors refer to different viral groups showed in the legend on the bottom.

The taxonomic composition of the ssDNA viral communities (PBI4s and PBOb are not shown here because of the limited number of affiliated ssDNA virus contigs, Table 2) was mostly comprised of the *Microviridae, Inoviridae, Geminiviridae* and *Circoviridae* families (Figure 3). *Microviridae* was the dominant ssDNA virus family in PBI2 (67.89%), PBI3 (73.59%), PBOs (57.09%), and PBI4b (87.55%). However, the majority of ssDNA viruses were *Inoviridae* (58.16%) in PBI1. Furthermore, according to the taxonomic ranking by species, the most abundant viral species amongst all samples was the marine gokushovirus (19.27–46.68%).

### Functional annotation of dsDNA viral communities in Prydz Bay

**Figure 4:**
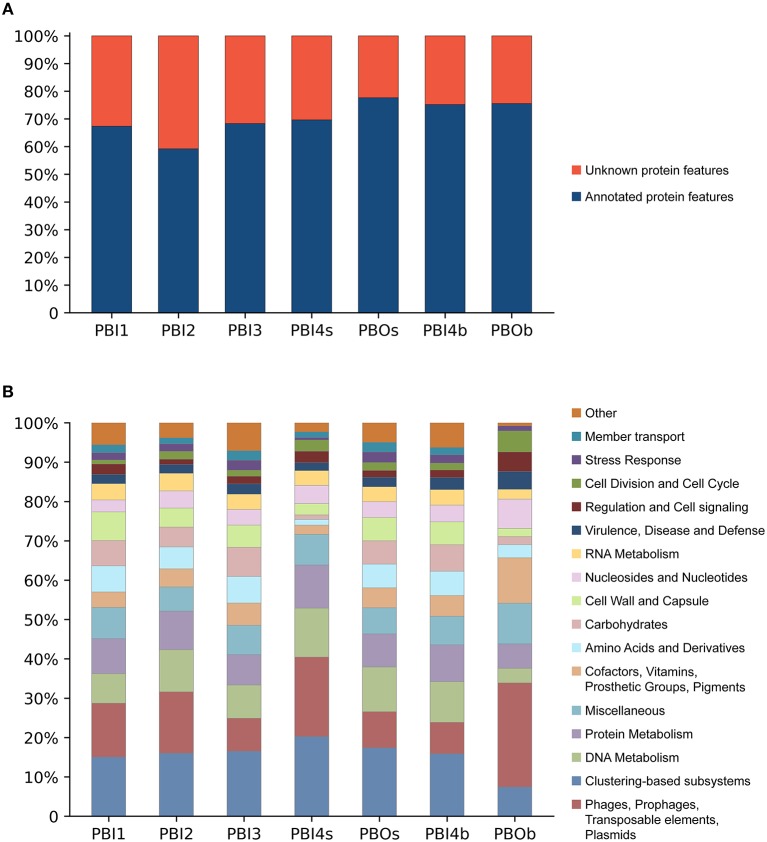
Functional analysis of annotated dsDNA viromes in the Prydz Bay, Antarctic. **(A)** Total predicted protein features of annotated dsDNA viromes and **(B)** the different subsystems categories of annotated protein features. Different colors refer to different functions showed in the legend on the right side.

The predicted protein features for dsDNA viral contigs and their functional distribution are presented in Figures 4A,B. For dsDNA viral contigs, 59.21–77.63% of the predicted proteins could be functionally annotated (Figure 4A). Five highly represented (>5%) functional categories were classified in PBI1, PBI2, PBI3, PBOs, and PBI4b, including “Clustering-based subsystems” (unknown function) (15.06–17.41%), “Phages, Prophages, Transposable elements, and Plasmids” (7.97–15.59%), followed by “DNA Metabolism” (7.51–11.36%), “Protein Metabolism” (7.72–9.83%), and “Miscellaneous” (6.12–7.91%). PBOb had the highest proportion (26.45%) of functional categories of “Phages, Prophages, Transposable elements, and Plasmids” amongst all samples (Figure 4B).

### Genome reconstruction

Reconstruction of dsDNA viral genomes from metagenomic sequences of each individual library was undertaken. 138,135 contigs, ranging from 301 to 130,439 bp, were uploaded to the MetaVir2 server for annotation and comparison with other publicly available viral genomes. The 52 long contigs, including five circular contigs, were reconstructed from the seven viral metagenomic samples, which ranged from 30 to 130.4 kb. The main characteristics of the 23 linear contigs longer than 40 kb and the five circular contigs are presented in Table 3. Among these long contigs, eighteen were assigned to Caudovirales and eight showed similarity to NCLDVs (*Mimiviridae* and *Phycodnaviridae*).

**Table 3:** Analysis of selected contigs in the Prydz Bay viromes.

**Contig ID**	**Length (bp)/type**	**No of ORF identified**	**No of ORF affiliated**	**Contig best BLAST hit affiliation**	**Terminase large subunit (TerL) affiliation**
PBI1_1_L	130,439/linear	126	24	*Myoviridae*; *Bacillus* phage 0305phi8-36	N/a
PBI1_2_L	51,889/linear	53	6	*Mimiviridae*; *Cafeteria* roenbergensis virus BV-PW1	N/a
PBI1_3_L	48,832/linear	53	12	*Podoviridae*; *Streptococcus* phage 315.1	N/a
PBI1_4_L	43,547/linear	46	5	*Myoviridae*; *Bacillus* virus G	N/a
PBI1_5_L	43,488/linear	33	10	*Siphoviridae*; *Streptomyces* phage Izzy	N/a
PBI2_1_L	64,194/linear	86	33	*Siphoviridae*; *Vibrio* phage VpKK5	*Siphoviridae*; *Streptomyces* phage mu1/6
PBI2_2_L	63,085/linear	65	13	*Mimiviridae*; *Megavirus* chiliensis	N/a
PBI2_3_L	45,901/linear	69	20	*Siphoviridae*; *Burkholderia* virus phi6442	*Siphoviridae*; *Burkholderia* virus phi6442
PBI2_4_L	45,864/linear	77	29	*Podoviridae*; *Bordetella* virus BPP1	N/a
PBI2_5_L	41,659/linear	53	6	*Phycodnaviridae*; *Aureococcus* anophagefferens virus	N/a
PBI2_6_L	40,234/linear	73	19	Unclassified dsDNA phages; *Sulfitobacter* phage NYA-2014a	Unclassified dsDNA phages; *Sulfitobacter* phage NYA-2014a
PBI3_1_L	95,664/linear	88	19	*Myoviridae*; *Bacillus* virus G	N/a
PBI3_2_L	83,424/linear	83	16	*Mimiviridae*; *Acanthamoeba polyphaga mimivirus*	N/a
PBI3_3_L	50,110/linear	59	8	*Phycodnaviridae*; *Aureococcus* anophagefferens virus	N/a
PBI3_4_L	46,251/linear	58	30	*Podoviridae*; *Enterobacteria* phage Min27	*Myoviridae*; *Clostridium* phage phiCD505
PBI3_5_L	43,879/linear	54	10	*Phycodnaviridae*; *Chrysochromulina* ericina virus	N/a
PBI4b_1_L	71,333/linear	68	10	*Mimiviridae*; *Acanthamoeba polyphaga mimivirus*	N/a
PBI4b_2_L	70,252/linear	67	14	*Myoviridae*; *Bacillus* virus G	N/a
PBI4b_3_L	68,047/linear	70	14	*Podoviridae*; *Streptococcus* phage 315.1	N/a
PBI4b_4_L	56,330/linear	69	13	*Phycodnaviridae*; *Chrysochromulina* ericina virus	N/a
PBI4b_5_L	51,529/linear	55	9	*Myoviridae*; *Escherichia* phage PBECO 4	N/a
PBI4b_6_L	42,027/linear	36	5	*Myoviridae*; *Bacillus* phage 0305phi8-36	N/a
PBI4b_7_L	40,544/linear	62	22	*Podoviridae*; *Edwardsiella* phage KF-1	Unclassified dsDNA phages; *Persicivirga* phage P12024L
PBI2_1_C	34,336/circular	58	25	*Siphoviridae*; *Flavobacterium* phage 11b	Unclassified phages; *Flavobacterium* phage Fpv5
PBI2_2_C	33,525/circular	40	27	Unclassified dsDNA phages; *Marinomonas* phage P12026	Unclassified dsDNA phages; *Marinomonas* phage P12026
PBI2_3_C	40,537/circular	63	23	*Podoviridae*; *Edwardsiella* phage KF-1	Unclassified dsDNA phages; *Persicivirga* phage P12024L
PBI3_1_C	41,598/circular	83	19	*Siphoviridae*; *Idiomarinaceae* phage Phi1M2-2	*Siphoviridae*; *Idiomarinaceae* phage Phi1M2-2
PBOs_1_C	40,652/circular	66	23	*Podoviridae*; *Burkholderia* virus Bcep22	*Podoviridae*; *Vibrio* phage PVA1

*Contigs longer than 40 kb or circular contigs are presented. N/a, no terminase large subunit (TerL) gene found*.

**Figure 5:**
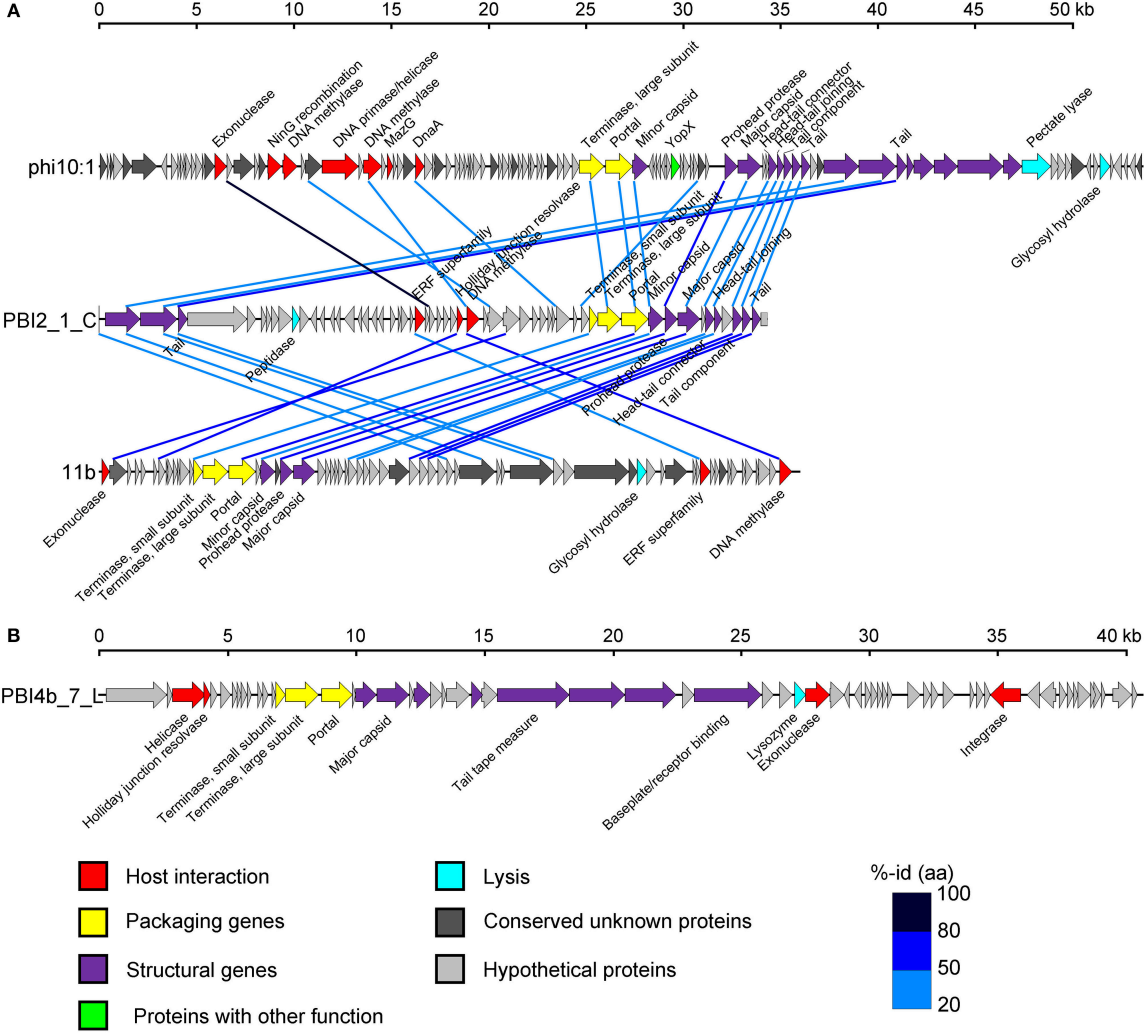
Genome analysis of putative complete viral genomes identified from the viral metagenomes in the Prydz Bay, Antarctic. **(A)** Comparisons of putative phage PBI2_1_C with *Cellulophaga* phage phi10:1 (NC_021802.1) and *Flavobacterium* phage 11b (NC_006356.2). **(B)** Genome map of putative phage PBI4b_7_L. Arrows represent predicted ORFs. The color code for predicted gene function is provided at the bottom of the figure. Homologous ORFs are connected by blue lines. %-id (aa) = amino acid identity.

An in-depth analysis was conducted on the two selected long contigs (PBI2_1_C and PBI4b_7_L) as well as for those that were identified as marine phages infecting *Bacteroidetes* bacteria. These two long contigs were 34,336 bp (PBI2_1_C) and 40,544 bp (PBI4b_1_L), with a G + C content of 35.30 and 35.00%, respectively. Circular contig PBI2_1_C contained 58 ORFs and represents a putative *Siphoviridae* phage. This is because 23 ORFs of PBI2_1_C showed a similarity to *Flavobacterium* phage 11b (NC_006356.2) and *Cellulophaga* phage phi10:1 (NC_021802.1) (Figure 5A), which are both marine members of the *Siphoviridae* family (Borriss et al., 2007; Holmfeldt et al., 2013). Most genes in PBI2_1_C with assigned function (fourteen of eighteen) encoded proteins related to viral structural and packaging. The terminase large subunit (TerL) gene showed a 40% amino acid similarity to the corresponding protein of *Flavobacterium* phage Fpv5 (NC_031921.1) and the major capsid protein (MCP) gene showed a 57% amino acid similarity to the gene of *Flavobacterium* phage 11b (NC_006356.2).

Linear contig PBI4b_7_L from the bottom sample probably also came from a member of the *Siphoviridae* family. Sixty-two ORFs were predicted within PBI4b_7_L, and no partially ORFs were detected at either end (Figure 5B). On the basis of the analysis of TerL, this phage could be classified as being related to the *Persicivirga* phage P12024L (NC_018272.1) (Kang et al., 2012); the structural ORFs were similar to *Cellulophaga* phage phi19:1 (NC_021799.1) (Holmfeldt et al., 2013) and *Polaribacter* phage P12002S (NC_028763.1) (Kang et al., 2015). All the matched phages' host bacterial strains belonged to the marine phylum *Bacteroidetes*.

### Relationship between viral community structure and environmental factors

**Figure 6:**
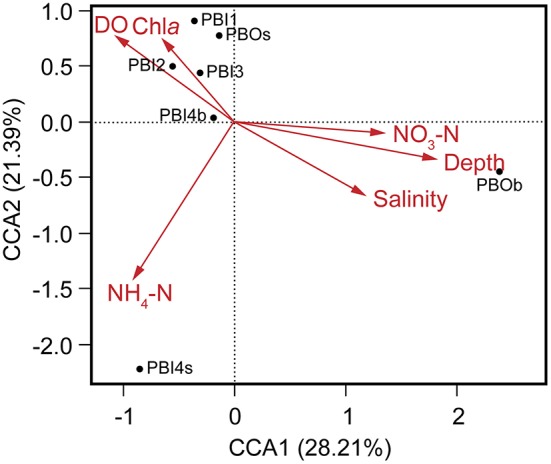
Canonical correspondence analysis of the relationship between the relative viral abundance of viral species and environmental factors. DO, dissolved oxygen; Chl*a*, chlorophyll *a*; NO_3_-N, nitrate; NH_4_-N, ammonium.

Multivariate regression analysis was used to determine the best predictor variables to explain the variation of the dsDNA viral community structure in the surface and deep sea of the Prydz Bay (Figure 6). The first CCA axis explained 28% of the total variability in the dsDNA viral community and the first two axes explained 50% of the total variability. The CCA showed that there were three clear groups of dsDNA viromes and the viromes of PBOb and PBI4s were clearly differentiated from the other samples. Most of surface dsDNA viromes (PBI1, PBI2, PBI3, and PBOs) were closely related to the concentrations of Chl*a* and DO. The dsDNA viromes of PBI4s were related to NH_4_-N, while the dsDNA viromes of PBOb were most closely related to the water depth and nutrient concentrations (Figure 6). The RDA results were similar to the results of the CCA (Figure S5).

### Comparison with the published aquatic viromes

In order to compare the Prydz Bay viromes with previously published data sets, 39 aquatic viromes and one soil virome of different environments: seawater (20), freshwater (15), hypersaline (3), estuarine water (1), and Antarctic hyperarid desert soil (1) were selected. The details of the selected viromes are shown in Table S3.

**Figure 7:**
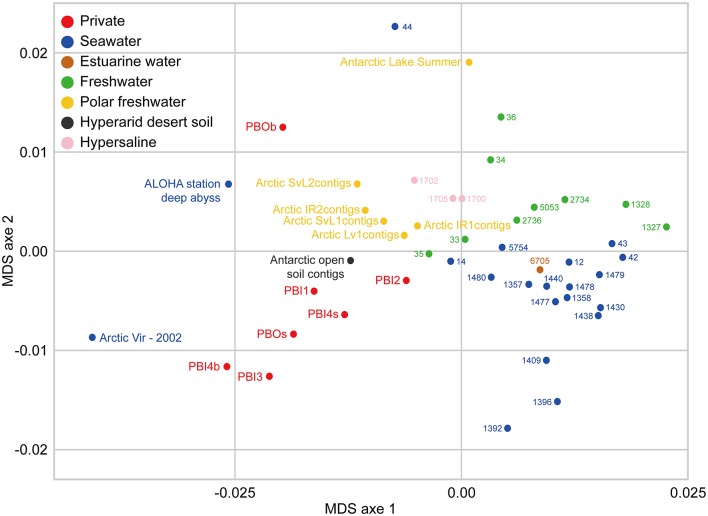
Comparison between DNA viromes in the Prydz Bay and other environmental DNA viromes based on k-mer frequency bias (tetranucleotides). Forty environmental DNA viromes available on MetaVir2 were selected from different kinds of environments, including seawater (20), freshwater (15), hypersaline (3), estuarine water (1), and Antarctic hyperarid desert soil (1). The number represented the project ID. The details of the selected viromes are shown in Table S3. Different colors refer to different environments showed in the legend on the top left.

Overall, the viromes appear to be clustered depending on the different sampling environments (Figure 7). The viromes could be classified into Prydz Bay viromes, marine viromes, freshwater viromes, polar freshwater viromes, and hypersaline viromes. Generally, the Prydz Bay viromes were closely related to the Arctic Ocean virome (Arctic Vir–2002; Arctic Ocean) (Angly et al., 2006), the Arctic freshwater viromes (Arctic contigs, Arctic lakes in Spitsbergen) (Cárcer et al., 2015) and the Antarctic desert soil virome (Antarctic open soil contigs; Miers Valley, Ross Dependency in eastern Antarctica) (Zablocki et al., 2014). However, the Prydz Bay viromes were quite different from the Antarctic freshwater virome (Antarctic Lake Summer; the ultra-oligotrophic freshwater lake Limnopolar in the WAP) (López-Bueno et al., 2009). Interestingly, the PBOb sample is quite similar to a deep sea virome from Station ALOHA (ALOHA station deep abyss), which is located in the North Pacific Gyre (22°45′N, 158°00′W).

## Discussion

Although marine viruses are recognized as the most abundant entities in the sea and play important roles in the marine ecosystems (Suttle, 2005), our knowledge about viral diversity, community structure and biogeography remains elusive, especially in the Southern Ocean. Currently, only the surface RNA and DNA viromes from the coastal waters of Palmer Station, Antarctic Peninsula, have been identified through metagenomic analysis (Brum et al., 2016; Miranda et al., 2016). Here, the first overview of the Prydz Bay viromes (including both surface and deep sea samples) is presented, showing that dsDNA viruses dominated the DNA viromes and Caudovirales and NCLDVs dominated the dsDNA viral population of the surface and bottom waters, respectively.

### Diversity of DNA viromes in Prydz Bay

In this study, with the exception of the PBI1 sample, the taxonomic analysis of affiliated DNA viromes showed that most viruses were classified as dsDNA viruses (Table 2, Figure S4), even when the viral DNA pool was randomly amplified using the MDA, which is known to increase the amplification of ssDNA molecules (Kim and Bae, 2011; Marine et al., 2014). It is suspected that ssDNA viruses were not generally abundant in Prydz Bay. In this study, as the DNA content obtained from the samples was quite limited, the DNase/RNase pretreatment of viral samples wasn't applied, which could bring in the viral and prophage DNA in the prokaryotic cells during the viral filtration and concentration (Steward and Preston, 2011; Winter et al., 2014). The viral concentrates were flash-frozen in liquid nitrogen and then stored at −80°C until processed (Brussaard, 2004; Winter et al., 2014). This viral storage approach is a typical protocol and verified as the best choice for viral conservation (Brussaard, 2004).

For dsDNA viruses, *Caudovirales*, which includes the families *Myoviridae, Siphoviridae*, and *Podoviridae*, was the dominant group (67.67–71.99%) in the surface waters of Prydz Bay (Figure 2). This result is similar to the findings of other marine viral metagenomic studies, such as Monterey Bay (65%), the Indian Ocean (95.3%), the Baltic Sea and the Antarctic Peninsula region of the Southern Ocean (~80%) (Steward and Preston, 2011; Williamson et al., 2012; Brum et al., 2016; Allen et al., 2017). *Caudovirales* were reported to infect a wide range of microbial hosts, including *Proteobacteria* and *Bacteroidetes*, which are dominant bacterial phyla in marine environments. Interestingly, *Cellulophaga* phages (phi38:1 and phi10:1) and *Flavobacterium* phage 11b, infecting the host strains *Cellulophaga baltica*, and *Flavobacterium hibernum*, respectively, were abundant in the surface dsDNA viromes of Prydz Bay. *Bacteroidetes* bacteria were abundant and active members of bacterial communities in Prydz Bay but are also present in other environments, such as Antarctic soil, surface, and deep ocean waters, etc. (Kirchman et al., 2005; Aislabie et al., 2006; González et al., 2007). These bacteria are thought to be important, as they are responsive to marine phytoplankton blooms and involved in degrading biopolymers, killing phytoplankton and recycling of phytoplankton-derived organic matter (Fernández-Gomez et al., 2013; Holmfeldt et al., 2013). In Prydz Bay, diatom and dinoflagellate blooms are often associated with the melting of the sea ice and a temperature rise (Zhang et al., 2014). In this study, the seawater samples were collected at the end time of the main summer phytoplankton bloom. Therefore, it is hypothesized that *Bacteroides* abundance increased during the phytoplankton bloom, which released organic matter into the surface waters and fueled the growth of *Bacteroides* (Fernández-Gomez et al., 2013; Holmfeldt et al., 2013). Subsequently, *Bacteroidetes* phages also became abundant in the dsDNA viromes of the Prydz Bay surface waters.

Interestingly, NCLDVs (*Phycodnaviridae, Mimiviridae*, and *Pandoraviridae*), were more abundant in the deep samples (18.74, 7.84, and 4.28% for PBOb and 8.80, 3.92, and 0.98% for PBI4b, respectively) than in the surface samples (3.54–8.66, 1.37–4.08, and 0.17–1.20%, respectively) (Figure 2). Within *Phycodnaviridae*, Prymnesiovirus (CeV and PgV contain large genomes of 510 and 560 kb, respectively), *Phaeovirus* (AaV), *Prasinovirus* (OlV2), and *Coccolithovirus* (EhV86) were dominant in PBOb. This study, however, did not provide the data to explain the high proportion of *Phycodnaviridae* in the deep sea as compared with that of that in the aphotic zone. However, based on other studies, possible explanations might include the long survival times and higher sedimentation rate of NCLDVs, sinking of the senescent algal bloom to the deep sea and subsequent release of high abundances of *Phycodnaviridae* (Danovaro et al., 2005; Borin et al., 2008; Corinaldesi et al., 2014; Antunes et al., 2015). In the present study, *Pandoraviridae* and *Mimiviridae* of NCLDVs were widespread in Prydz Bay. However, PBOb had the highest proportion of *Pandoraviridae* (4.28%) and *Mimiviridae* (including ApMoV and ApMiV infecting *A*. polyphaga, 5.93%) and there was also a relatively high proportion of *Pandoraviridae* and *Mimiviridae* in PBI4b (2.46%); these were higher than in the surface seawater (Tables S1, S2). Both *Pandoraviridae* and *Acanthamoeba polyphaga* viruses, which are giant viruses within NCLDVs, infect amoebae and are also known as “ancient virus” (Yutin and Koonin, 2012; Legendre et al., 2014). In this study, the viral metagenomic samples were collected according to the traditional viral concentration methods, which combined the 0.22 μm pore size filtration and the tangential-flow filtration, which is not specifically considered for NCLDVs (Hurwitz and Sullivan, 2013; Winter et al., 2014). The proportion of NCLDVs in the viral metagenomic samples might have been underestimated by the using of the 0.22 μm pore size filtration.

In PBI4b, the most abundant viral species was *Psychrobacter* phage Psymv2 (NC_023734.1) (4.02%). This is a temperate bacteriophage from the Antarctic Dry Valleys (78°05′S, 163°45′E, Miers Valley in the McMurdo Dry Valleys, South Victoria Land, Antarctica) soil isolate *Psychrobacter* sp. MV2 (Meiring et al., 2012). The host bacteria strains of this phage include some members of the genus *Psychrobacter*, which have been isolated from a wide range of habitats, including surface and deep sea waters, deep sea sediments and soil, especially from the Antarctic region, and are also widespread in cold Antarctic environments (Romanenko et al., 2002; Zhang et al., 2007; Meiring et al., 2012). Data presented here suggest that the Prydz Bay seawater and the McMurdo Dry Valleys of eastern Antarctic soil might contain similar viruses and that there are also a large number of cold-adapted *Psychrobacter* bacteria in the bottom waters of Prydz Bay. In the future, it will be necessary to analyze the host community structure simultaneously to verify the speculated relationship between the virus and the viral potential host cells.

Of the ssDNA viruses, marine gokushovirus (19.27–46.68%) were ubiquitous in Prydz Bay. Gokushoviruses belong to the subfamily *Gokushovirinae*, within the *Microviridae*, which are well represented in sequences found in metagenomic databases (Labonté and Suttle, 2013). These metagenomic studies indicate that Gokushoviruses are genetically diverse and widespread members of marine ssDNA viral communities; they are also ubiquitous in the Prydz Bay.

It should be noted that the viral diversity and community structure found in Prydz Bay was determined from the affiliated contigs, which only represented a small fraction of the possible viral contigs (10.35–14.92%, Figure S3). The unaffiliated contigs contain a high proportion of unknown genes, which are most likely linked to environmentally-specific viruses from the Prydz Bay ecosystem. These findings support the hypothesis that much of the global marine viral diversity remains uncharacterized. Similar results have been reported from other published aquatic DNA viromes (Breitbart et al., 2002; Steward and Preston, 2011; Williamson et al., 2012; Winter et al., 2014; Brum et al., 2016; Cai et al., 2016; Roux et al., 2016b; Skvortsov et al., 2016). Because there are too many uncultured viral species in the marine environments, the reference genome database for viral metagenomic analysis is still far from complete and an extraordinary amount of uncharacterized viral “dark matter” still exists in seawater. Although metagenomic approaches have allowed the assembly of long contigs and the complete analysis of predicted proteins instead of short reads, most ORFs are still unknown. In future, the incorporation of robust viral genomic information from viral isolates and metagenomic data into the viral taxonomy will be a major contribution to research on viral diversity and will lead to a better understanding of the ecology, history, and impact of global viromes (Simmonds et al., 2017).

### Novel assembled genomes

Two novel genomes (PBI2_1_C in circular and PBI4b_7_L in linear) were assembled and selected for detailed analysis from the DNA viral metagenomic libraries; these will contribute to our knowledge of the marine *Bacteroidetes* phage genomes (Miranda et al., 2016). The genomic structural and TerL phylogenetic analysis of PBI2_1_C showed the highest relationship with the viral isolates (*Flavobacterium* phage 11b and *Cellulophaga* phage phi10:1) from the *Cellulophaga* and *Flavobacterium* host cells, which belong to the phylum *Bacteroidetes*. *Flavobacterium* phage 11b and *Cellulophaga* phage phi10:1. These were originally isolated from Arctic sea-ice and the strait of Öresund, between Sweden and Denmark (Holmfeldt et al., 2013), and had a high genome similarity to each other. *Flavobacterium* phage 11b belongs to the tail dsDNA phage family *Siphoviridae*. The comparative genome analysis of PBI2_1_C, *Flavobacterium* phage 11b and *Cellulophaga* phage phi10:1 showed that they shared similar structural modules at the gene level (Figure 5A). The high genome similarity between these two isolated phages and the assembled Antarctic viral contig shows that Antarctic and Arctic waters might contain similar viruses, potentially infecting cold-adapted microorganisms.

### Comparison between the Prydz Bay viromes and the published aquatic viromes

In this study, some interesting results were found by comparing the viromes in Prydz Bay with other published viromes. In the MDS biplot, the Prydz Bay viromes were similar to the Arctic freshwater virome (Arctic contigs) and the Arctic Ocean viromes (Arctic Vir–2002) but they were different from the Antarctic freshwater virome (Antarctic Lake Summer). The Arctic freshwater viral community was reported to be different from that of the Arctic Ocean, and shared viral taxonomic communities with Antarctic freshwater ecosystems (though with very low fine-grain genetic overlap) (Cárcer et al., 2015). To some extent, the results presented here demonstrated that there were correlations between the dsDNA viral communities in the Antarctic and the Arctic. It also reflected that there was a complex relationship between polar freshwater and marine viromes.

It was also found that the Prydz Bay viromes were closely related to the Antarctic desert soil virome (Antarctic open soil contigs; Miers Valley) (Zablocki et al., 2014). A large number of *Psychrobacter* phage Psymv2, which was isolated from the Miers Valley (Meiring et al., 2012), was also found in the Prydz Bay viromes. These results provide some evidence for the study of the origin and distribution of viruses in the Antarctic region and the relationship between the marine and Antarctic desert environments.

**Figure 8:**
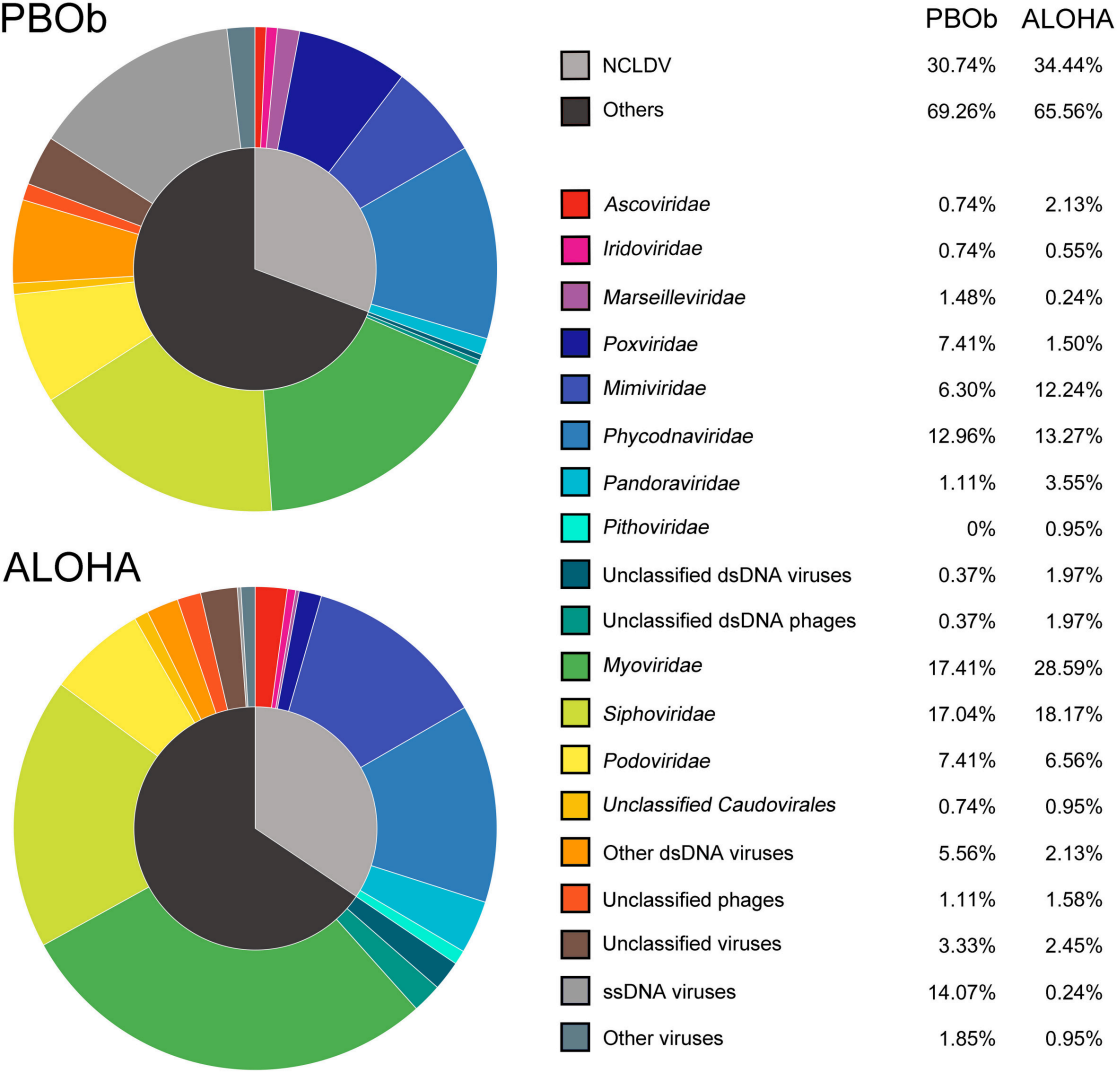
Comparison of viral taxonomic compositions between PBOb and deep sea sample of ALOHA station. The non-normalized taxonomic composition data based on contigs best BLAST hit (threshold of 50 on the BLAST bit score) of PBOb (Project ID 8183) and ALOHA station deep abyss (Project ID 3816) were downloaded from MetaVir2 server. Different colors refer to different viral groups showed in the legend on the right side.

The dsDNA viral community in the bottom waters of Prydz Bay (PBOb) were quite different from those of the surface water viromes (Figures 2, 6). In PBOb, the *Phycodnaviridae* and giant viruses *Mimiviridae*, which were within NCLDVs, were abundant (Figure 2). Interestingly, such a large proportion of NCLDVs in the deep waters (PBOb, >3,000 mbs, 30.74%) was similar to the one deep sea virome at ALOHA (34.44%, Figure 8). Knowledge of deep sea Antarctic viromes is very limited and here possible mechanisms for the dominance of NCLDVs in the deep sea viromes are suggested. Firstly, it is possible that the phytoplankton blooms in the surface waters of Prydz Bay induced high infection rates of the phytoplankton and protists by NCLDVs; secondly, the infected algae and protists sank to the deep sea, subsequently lysing and releasing the NCLDVs into the deep water.

### Methodological considerations about the whole genome amplification (WGA)

The MDA, one of the most commonly used WGA methods was used to get sufficient DNA for viral metagenomic sequencing in this study. Though MDA is known to result in amplification biases and to increase the amplification of ssDNA molecules (Kim and Bae, 2011; Marine et al., 2014), MDA has been successfully used in many viral metagenomic studies in different environments (Angly et al., 2006; Labonté and Suttle, 2013; Zablocki et al., 2014; Roux et al., 2016b).

To avoid the influences caused by the MDA amplification, several measures have been chosen, including: (1). The Qiagen REPLI-g kit, which was proved to be the ideal choice for the WGA kit, was used in this study (Thoendel et al., 2017); (2) This study paid primary attention on the dsDNA viral communities that were less affected by MDA; (3) For comparison with other published aquatic viromes, a viral metagenomic study that carried out MDA and performed MetaVir2 for the comparisons among different samples were referred to Roux et al. (2016b). In addition, a recent study reported that the bias induced by WGA has only a limited impact on the beta diversity of human saliva viromes (Parras-Moltó et al., 2018), hence MDA might have a smaller than expected impact on the comparisons among different samples. Though MDA could be considered as an acceptable method for the study of viral community, the direct sequencing method without amplification is still the first choice to illustrate the actual viral community in the future, especially with the rapid technological progress on the high-throughput sequencing.

## Conclusion

This study describes the surface and deep sea viral community structure of Prydz Bay, Antarctic, based on a metagenomic analysis and its relationship with environmental factors. dsDNA viromes dominated the local DNA viral community structure; Caudovirales and NCLDVs were the most abundant dsDNA viromes at the surface and in the deep sea, respectively. Although the reason for the abundance of NCLDVs in the deep sea is not yet known, data presented here suggest that NCLDVs are an abundant component of deep sea viral communities around the Antarctic. In future, the simultaneously study of the 16S rRNA, 18S rRNA genes, and viral profiles of the same seawater samples should provide an insight into the detailed relationship between viruses and their possible hosts in the Antarctic.

## Author contributions

MW, ZG, and YL designed this study. MW and JW performed the experiments. Data were analyzed by ZG in collaboration with YL, QY, JX, and YJ. ZG and YL wrote the manuscript. AM, YJ, XZ, SY, CG, JH, and HS contributed to writing by providing suggestions and helping with the revisions. All authors reviewed and approved the final version of the manuscript.

### Conflict of interest statement

The authors declare that the research was conducted in the absence of any commercial or financial relationships that could be construed as a potential conflict of interest.

## Supplementary material

Figure S1 Workflow for contigs assembly and analyses.

Figure S2 Cluster analysis of the environmental factors of samples.

Figure S3 Affiliated and unaffiliated contigs in Prydz Bay. Affiliated contigs: contigs were assigned to be of viral origin; affiliated dsDNA viruses contigs: contigs were assigned to be of dsDNA viruses.

Figure S4 Relative abundances of viral contigs after normalization (DNA-RPKM).

Figure S5 Redundancy analysis of the relationship between the relative viral abundance of viral species and environmental factors. DO, dissolved oxygen; Chl*a*, chlorophyll *a*; NO_3_-N, nitrate; NH_4_-N, ammonium.

Table S1 Taxonomic viral composition based on LCA affiliation for viral contigs via MetaVir2. Relative quantities expressed as DNA-RPKM numbers and percentage.

Table S2 Specific viral composition based on host taxonomies. Relative quantities expressed as percentage.

Table S3 Detailed information of selected environmental DNA viromes available on MetaVir2.
