# Supplementary figures and images for: Viral Diversity and Its Relationship With Environmental Factors at the Surface and Deep Sea of Prydz Bay, Antarctica
Source: Front Microbiol. 2018 Dec 3;9:2981. doi: 10.3389/fmicb.2018.02981 (PMC6287040; doi:10.3389/fmicb.2018.02981)

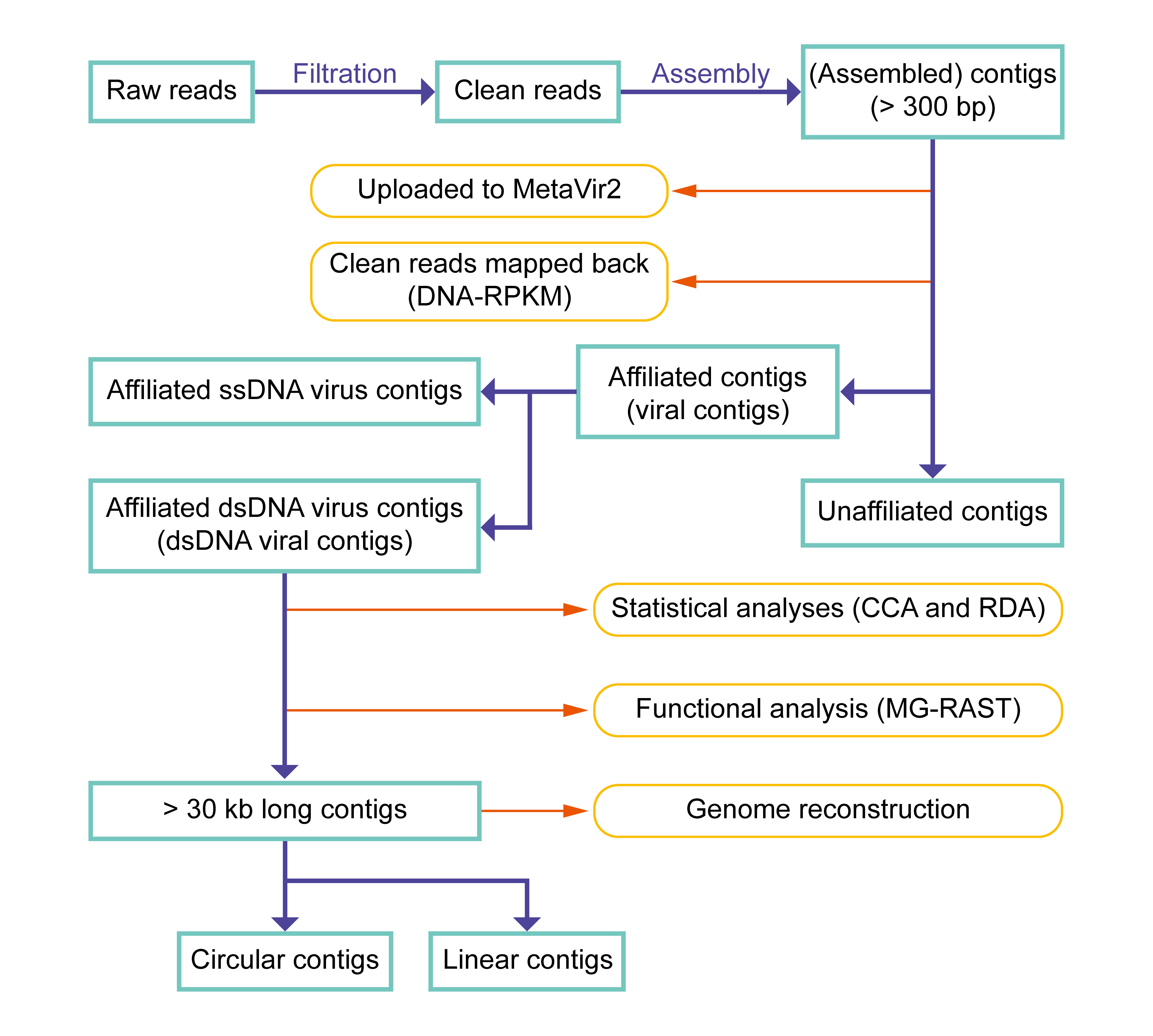

Supplement: Figure S1 — Workflow for contigs assembly and analyses. [file Image_1.TIF]

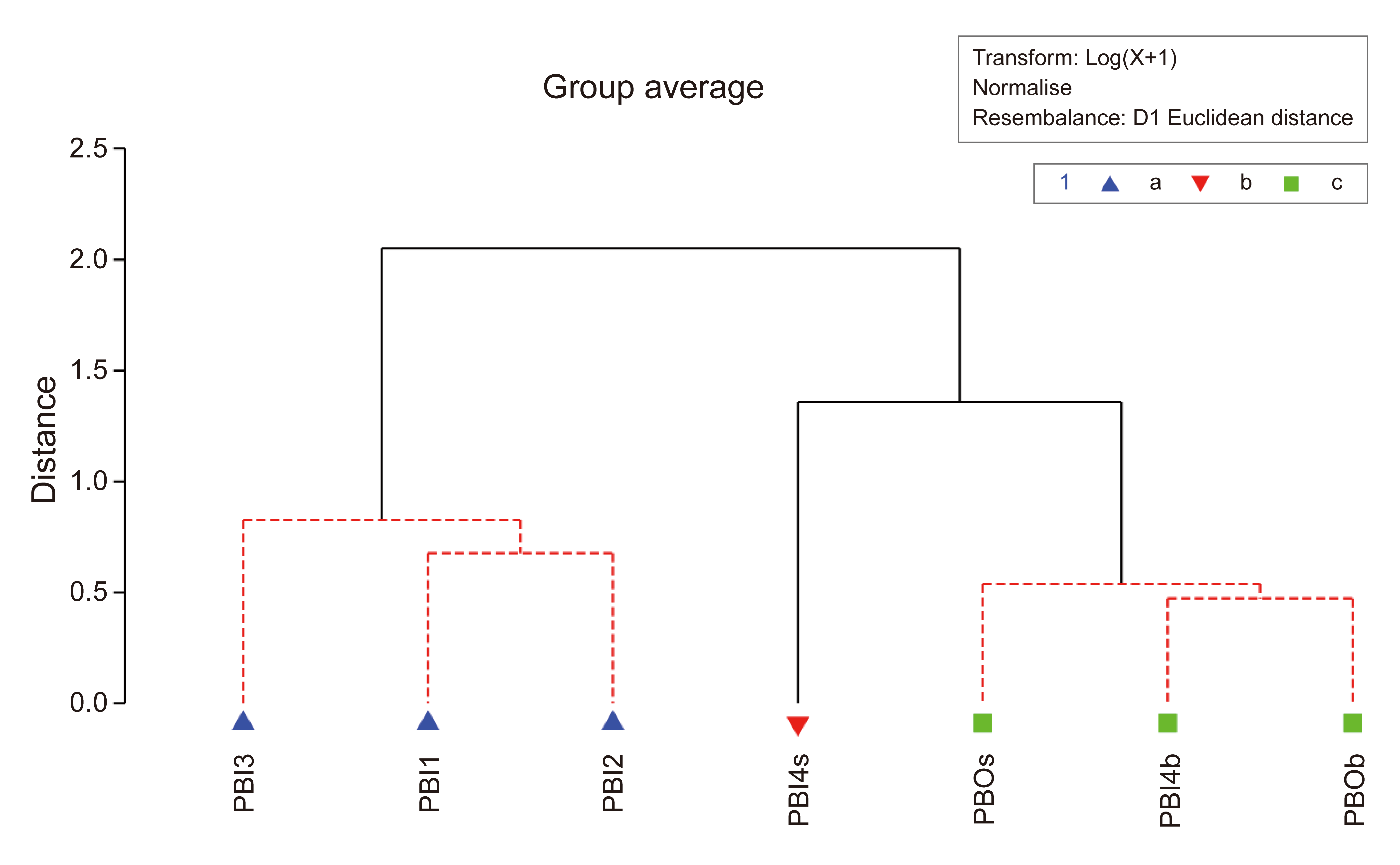

Supplement: Figure S2 — Cluster analysis of the environmental factors of samples. [file Image_2.TIF]

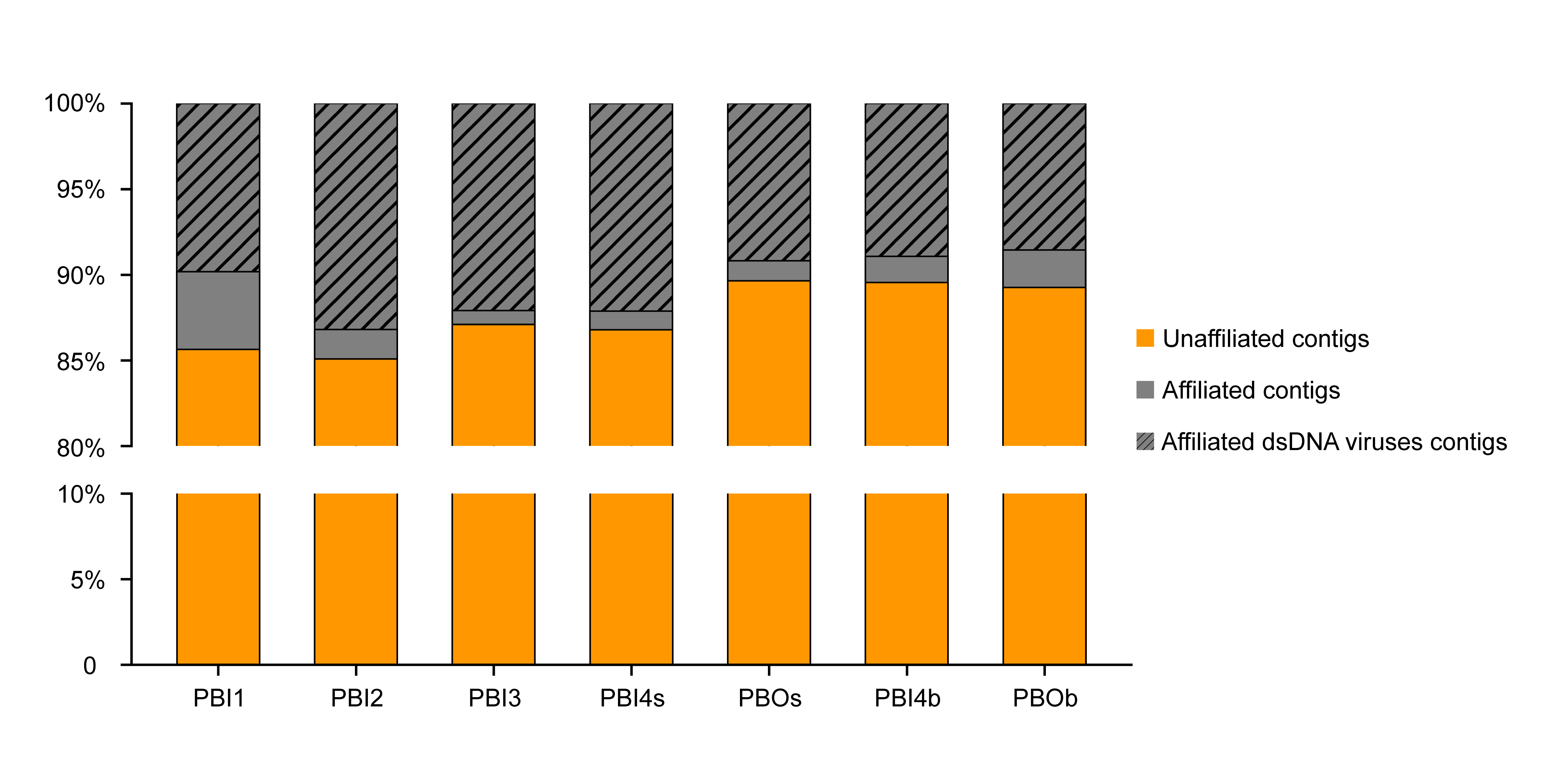

Supplement: Figure S3 — Affiliated and unaffiliated contigs in Prydz Bay. Affiliated contigs: contigs were assigned to be of viral origin; affiliated dsDNA viruses contigs: contigs were assigned to be of dsDNA viruses. [file Image_3.TIF]

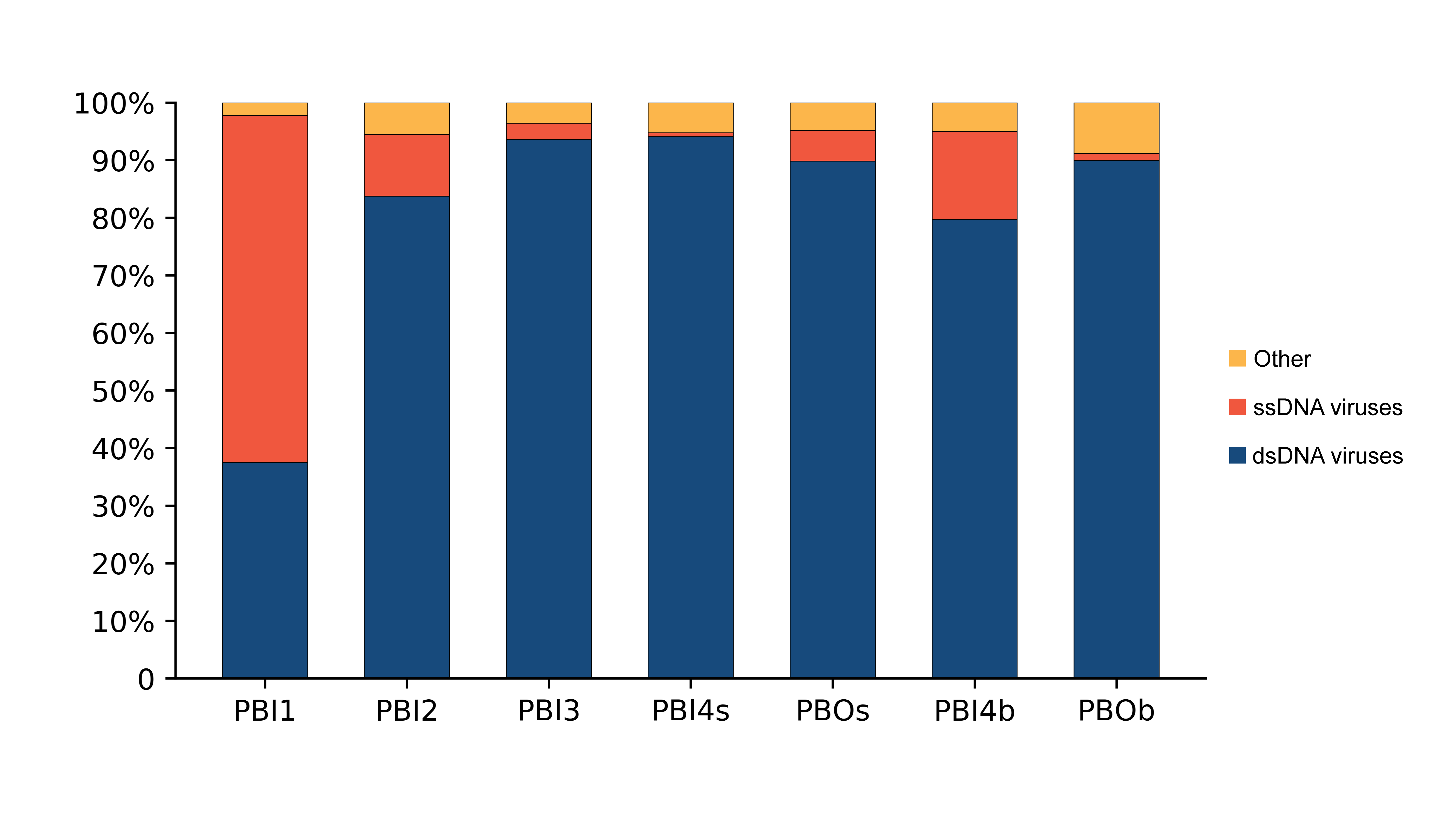

Supplement: Figure S4 — Relative abundances of viral contigs after normalization (DNA-RPKM). [file Image_4.TIF]

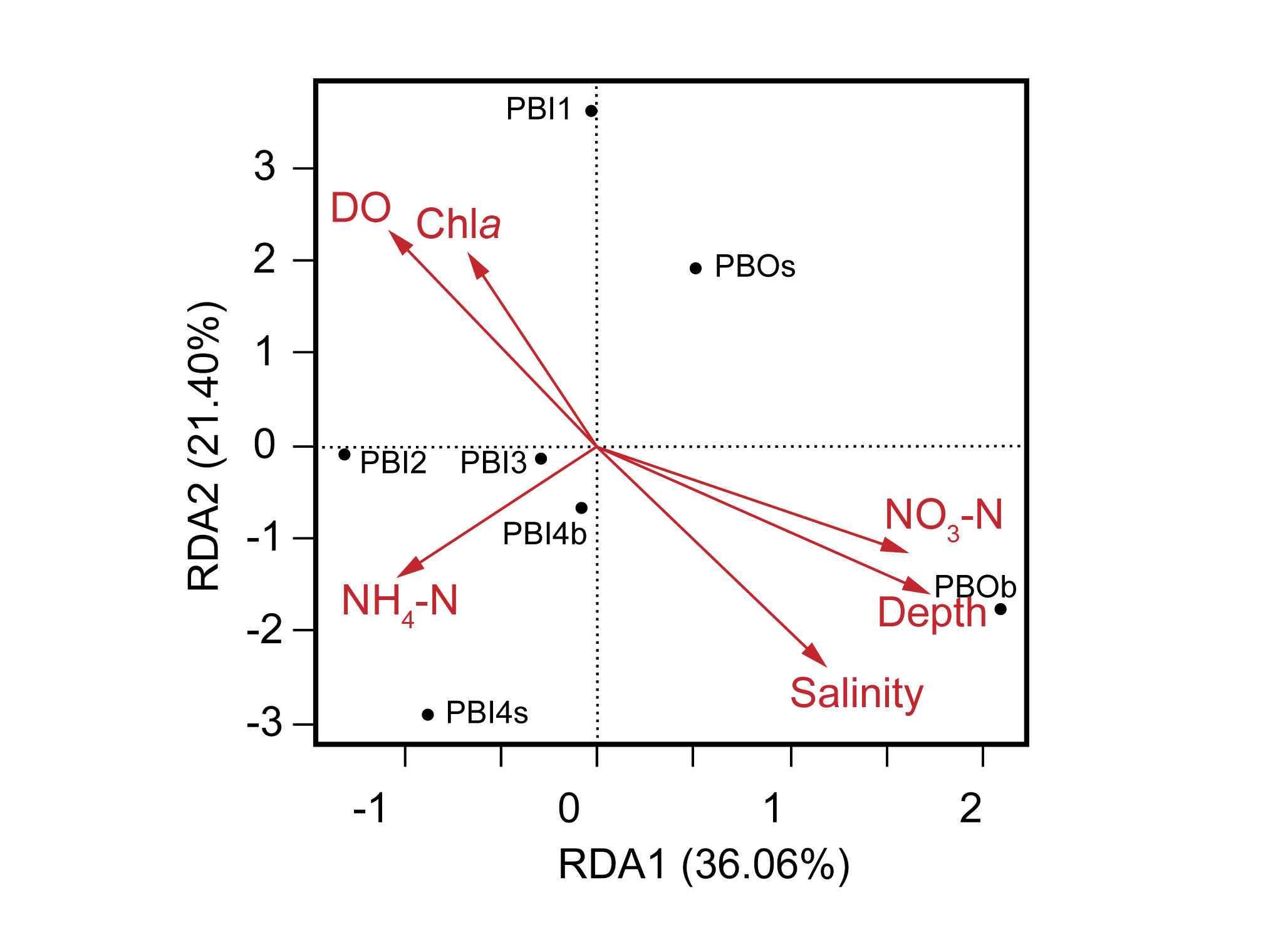

Supplement: Figure S5 — Redundancy analysis of the relationship between the relative viral abundance of viral species and environmental factors. DO, dissolved oxygen; Chla, chlorophyll a; NO3-N, nitrate; NH4-N, ammonium. [file Image_5.TIF]
